# Fraxinellone

**DOI:** 10.1107/S1600536811018393

**Published:** 2011-05-20

**Authors:** Hong-Mei Gu, Hao Xu, Zhao-Zhao Zhong, Hai-Liang Zhu, Qing-Shan Li

**Affiliations:** a Jiangsu Chiatai Tianqing Pharmaceutical Co. Ltd, Nanjing 210042, People’s Repulic of China, and, State Key Laboratory of Pharmaceutical Biotechnology, Nanjing University, Nanjing 210093, People’s Republic of China

## Abstract

In the title compound, C_14_H_16_O_3_ [systematic name: (3R*,3aR*)-3-(3-furan­yl)-3a,7-dimethyl-3a,4,5,6-tetra­hydro-2-benzofuran-1(3*H*)-one], the pendant methyl and furan groups attached to the stereogenic centres lie to the same side of the fused ring system. The dihedral angle between the five-membered rings is 74.8 (2)°; the fused five-membered ring adopts a twisted conformation. In the crystal, mol­ecules are linked by weak C—H⋯O inter­actions, which generate [100] chains.

## Related literature

For background to fraxinellone and its biological activity, see: Kim *et al.* (2009 ▶); Sun *et al.* (2009 ▶); Liu *et al.* (2009 ▶). For standard bond lengths, see: Allen *et al.* (1987 ▶).
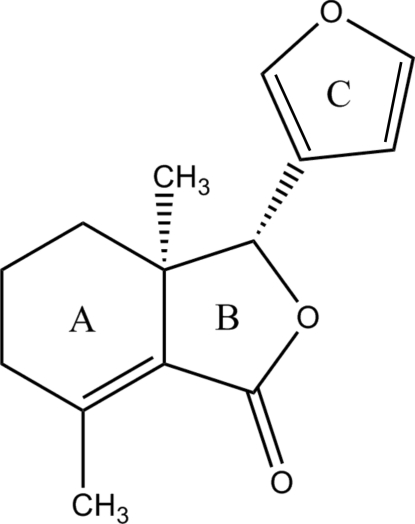


## Experimental

### 

#### Crystal data

C_14_H_16_O_3_
*M* *_r_* = 232.27
Orthorhombic, 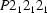

*a* = 5.940 (3) Å
*b* = 12.661 (6) Å
*c* = 15.921 (7) Å
*V* = 1197.3 (9) Å^3^
*Z* = 4
Mo *K*α radiation
μ = 0.09 mm^−1^
*T* = 298 K
0.20 × 0.20 × 0.18 mm

#### Data collection

Bruker SMART CCD diffractometer
Absorption correction: multi-scan (*SADABS*; Bruker, 2000 ▶) *T*_min_ = 0.982, *T*_max_ = 0.984
10397 measured reflections
1733 independent reflections
1331 reflections with *I* > 2σ(*I*)
*R*_int_ = 0.049

#### Refinement

*R*[*F* ^2^ > 2σ(*F* ^2^)] = 0.046
*wR*(*F* ^2^) = 0.113
*S* = 1.07
1733 reflections
156 parameters
H-atom parameters constrained
Δρ_max_ = 0.12 e Å^−3^
Δρ_min_ = −0.17 e Å^−3^

### 

Data collection: *SMART* (Bruker, 2000 ▶); cell refinement: *SAINT* (Bruker, 2000 ▶); data reduction: *SAINT*; program(s) used to solve structure: *SHELXL97* (Sheldrick, 2008 ▶); program(s) used to refine structure: *SHELXTL* (Sheldrick, 2008 ▶); molecular graphics: *SHELXTL*; software used to prepare material for publication: *SHELXTL*.

## Supplementary Material

Crystal structure: contains datablocks global, I. DOI: 10.1107/S1600536811018393/hb5863sup1.cif

Structure factors: contains datablocks I. DOI: 10.1107/S1600536811018393/hb5863Isup2.hkl

Supplementary material file. DOI: 10.1107/S1600536811018393/hb5863Isup3.cml

Additional supplementary materials:  crystallographic information; 3D view; checkCIF report

## Figures and Tables

**Table 1:** Hydrogen-bond geometry (Å, °)

*D*—H⋯*A*	*D*—H	H⋯*A*	*D*⋯*A*	*D*—H⋯*A*
C8—H8⋯O1^i^	0.98	2.58	3.510 (3)	158

Symmetry code: (i) 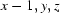.

**Fig. 1.**
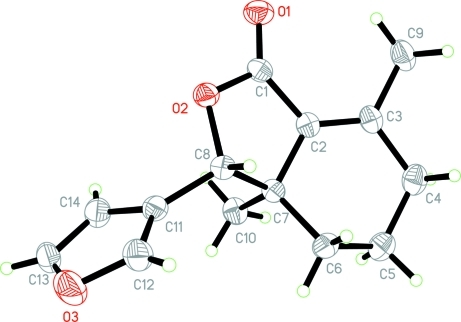
The molecular structure of (I) showing 30% probability displacement ellipsoids.

## supplementary crystallographic information

### Comment

There has been much research interest in fraxinellone due to its biological activities (Kim *et al.* (2009); Sun *et al.* (2009); Liu *et al.* (2009)). In this work, we report here the crystal structure of the title compound, (I). In (I), all bond lengths are within normal ranges (Allen *et al.*, 1987) (Fig. 1). The dihedral angle between the C1—C2—C7—C8—O2 and C12—C11—C14—C13—O3 rings is 74.8 (2)°.

### Experimental

In order to extract the fraxinellone with bioactivity containing in Dictamnus dasycarpus Turks, 100 g/*L* milk of lime wetting plant material, was extracted by using refluent extract method with petroleum ether as a solvent. The residue was separated with methanol and petroleum ether, and recrystallized in methanol. It was further purified on a silica gel column. Crystals suitable for X-ray structure analysis were obtained by slow evaporation of a solution in methanol at room temperature.

### Refinement

Anomalous dispersion was negligible and Friedel pairs were merged before refinement. All H atoms were positioned geometrically (C—H = 0.93 Å for the aromatic H atoms and C—H = 0.96 Å for the aliphatic H atoms) and were refined as riding, with *U*_iso_(H) = 1.2*U*_eq_(C) and *U*_iso_(H) = 1.2*U*_eq_(N).

### Crystal data

**Table:**

C_14_H_16_O_3_	*F*(000) = 496
*M**_r_* = 232.27	*D*_x_ = 1.288 Mg m^−^^3^
Orthorhombic, *P*2_1_2_1_2_1_	Mo *K*α radiation, λ = 0.71073 Å
Hall symbol: P 2ac 2ab	Cell parameters from 25 reflections
*a* = 5.940 (3) Å	θ = 9–12°
*b* = 12.661 (6) Å	µ = 0.09 mm^−^^1^
*c* = 15.921 (7) Å	*T* = 298 K
*V* = 1197.3 (9) Å^3^	Block, colorless
*Z* = 4	0.20 × 0.20 × 0.18 mm

### Data collection

**Table:**

Bruker SMART CCD diffractometer	1733 independent reflections
Radiation source: fine-focus sealed tube	1331 reflections with *I* > 2σ(*I*)
graphite	*R*_int_ = 0.049
φ and ω scans	θ_max_ = 28.5°, θ_min_ = 2.1°
Absorption correction: multi-scan (*SADABS*; Bruker, 2000)	*h* = −7→7
*T*_min_ = 0.982, *T*_max_ = 0.984	*k* = −17→16
10397 measured reflections	*l* = −19→21

### Refinement

**Table:**

Refinement on *F*^2^	Primary atom site location: structure-invariant direct methods
Least-squares matrix: full	Secondary atom site location: difference Fourier map
*R*[*F*^2^ > 2σ(*F*^2^)] = 0.046	Hydrogen site location: inferred from neighbouring sites
*wR*(*F*^2^) = 0.113	H-atom parameters constrained
*S* = 1.07	*w* = 1/[σ^2^(*F*_o_^2^) + (0.0425*P*)^2^ + 0.1558*P*] where *P* = (*F*_o_^2^ + 2*F*_c_^2^)/3
1733 reflections	(Δ/σ)_max_ < 0.001
156 parameters	Δρ_max_ = 0.12 e Å^−^^3^
0 restraints	Δρ_min_ = −0.17 e Å^−^^3^

### Special details

**Table:**

Geometry. All e.s.d.'s (except the e.s.d. in the dihedral angle between two l.s. planes) are estimated using the full covariance matrix. The cell e.s.d.'s are taken into account individually in the estimation of e.s.d.'s in distances, angles and torsion angles; correlations between e.s.d.'s in cell parameters are only used when they are defined by crystal symmetry. An approximate (isotropic) treatment of cell e.s.d.'s is used for estimating e.s.d.'s involving l.s. planes.
Refinement. Refinement of *F*^2^ against ALL reflections. The weighted *R*-factor *wR* and goodness of fit *S* are based on *F*^2^, conventional *R*-factors *R* are based on *F*, with *F* set to zero for negative *F*^2^. The threshold expression of *F*^2^ > σ(*F*^2^) is used only for calculating *R*-factors(gt) *etc*. and is not relevant to the choice of reflections for refinement. *R*-factors based on *F*^2^ are statistically about twice as large as those based on *F*, and *R*- factors based on ALL data will be even larger.

### Fractional atomic coordinates and isotropic or equivalent isotropic displacement parameters (Å^2^)

**Table:**

	*x*	*y*	*z*	*U*_iso_*/*U*_eq_	
O1	0.5425 (3)	−0.10394 (15)	0.61505 (12)	0.0558 (5)	
O2	0.2594 (3)	0.00000 (13)	0.65310 (9)	0.0423 (4)	
O3	−0.2584 (5)	0.24662 (18)	0.72681 (14)	0.0806 (8)	
C1	0.3749 (4)	−0.05682 (18)	0.59460 (15)	0.0391 (5)	
C2	0.2628 (4)	−0.04409 (17)	0.51292 (14)	0.0377 (5)	
C3	0.2806 (5)	−0.10573 (18)	0.44505 (15)	0.0428 (6)	
C4	0.1270 (5)	−0.0894 (2)	0.37091 (17)	0.0571 (7)	
H4A	0.0334	−0.1517	0.3649	0.069*	
H4B	0.2190	−0.0840	0.3208	0.069*	
C5	−0.0254 (5)	0.0067 (2)	0.37495 (17)	0.0583 (7)	
H5A	−0.1572	−0.0058	0.3404	0.070*	
H5B	0.0539	0.0671	0.3520	0.070*	
C6	−0.1009 (4)	0.0323 (2)	0.46465 (16)	0.0488 (6)	
H6A	−0.1916	0.0960	0.4645	0.059*	
H6B	−0.1925	−0.0251	0.4862	0.059*	
C7	0.1041 (4)	0.04793 (17)	0.52105 (13)	0.0355 (5)	
C8	0.0486 (4)	0.03908 (17)	0.61578 (14)	0.0364 (5)	
H8	−0.0667	−0.0156	0.6228	0.044*	
C9	0.4404 (5)	−0.1978 (2)	0.4381 (2)	0.0599 (8)	
H9A	0.5589	−0.1806	0.3995	0.090*	
H9B	0.3603	−0.2587	0.4181	0.090*	
H9C	0.5037	−0.2127	0.4923	0.090*	
C10	0.2300 (5)	0.15067 (19)	0.50129 (15)	0.0466 (6)	
H10A	0.3577	0.1572	0.5379	0.070*	
H10B	0.1311	0.2097	0.5096	0.070*	
H10C	0.2802	0.1495	0.4440	0.070*	
C11	−0.0265 (4)	0.13529 (19)	0.66193 (14)	0.0416 (6)	
C12	−0.2403 (6)	0.1508 (2)	0.68932 (16)	0.0563 (7)	
H12	−0.3574	0.1026	0.6833	0.068*	
C13	−0.0487 (6)	0.2921 (2)	0.72441 (18)	0.0579 (8)	
H13	−0.0116	0.3577	0.7467	0.069*	
C14	0.0930 (6)	0.2275 (2)	0.68509 (17)	0.0542 (7)	
H14	0.2445	0.2406	0.6746	0.065*	

### Atomic displacement parameters (Å^2^)

**Table:**

	*U*^11^	*U*^22^	*U*^33^	*U*^12^	*U*^13^	*U*^23^
O1	0.0482 (11)	0.0579 (10)	0.0612 (11)	0.0135 (10)	−0.0039 (10)	0.0022 (9)
O2	0.0457 (9)	0.0438 (8)	0.0372 (8)	0.0068 (8)	0.0002 (8)	0.0015 (7)
O3	0.101 (2)	0.0835 (16)	0.0576 (12)	0.0376 (17)	0.0124 (14)	−0.0099 (11)
C1	0.0405 (13)	0.0328 (11)	0.0441 (13)	−0.0013 (11)	0.0046 (11)	0.0044 (10)
C2	0.0383 (13)	0.0356 (11)	0.0391 (12)	−0.0052 (11)	0.0047 (11)	0.0025 (9)
C3	0.0415 (14)	0.0389 (12)	0.0480 (14)	−0.0062 (11)	0.0090 (12)	−0.0047 (10)
C4	0.0623 (18)	0.0652 (16)	0.0438 (15)	−0.0026 (16)	0.0025 (14)	−0.0105 (13)
C5	0.0666 (19)	0.0645 (16)	0.0438 (14)	0.0014 (16)	−0.0078 (14)	−0.0009 (13)
C6	0.0476 (15)	0.0539 (15)	0.0449 (14)	0.0050 (13)	−0.0071 (12)	−0.0008 (12)
C7	0.0375 (12)	0.0348 (11)	0.0342 (11)	0.0006 (10)	0.0034 (10)	0.0013 (9)
C8	0.0351 (12)	0.0373 (11)	0.0369 (11)	0.0000 (10)	0.0024 (10)	0.0023 (9)
C9	0.0583 (19)	0.0508 (15)	0.0708 (19)	0.0022 (15)	0.0055 (16)	−0.0183 (14)
C10	0.0561 (16)	0.0385 (11)	0.0451 (13)	−0.0066 (13)	0.0066 (14)	0.0048 (10)
C11	0.0467 (15)	0.0454 (13)	0.0326 (11)	0.0058 (12)	0.0009 (11)	0.0024 (10)
C12	0.0607 (18)	0.0642 (16)	0.0439 (14)	0.0099 (16)	0.0075 (16)	−0.0036 (13)
C13	0.082 (2)	0.0455 (14)	0.0465 (15)	0.0112 (16)	−0.0031 (16)	−0.0107 (12)
C14	0.0634 (19)	0.0508 (14)	0.0485 (15)	−0.0008 (15)	−0.0041 (15)	−0.0052 (12)

### Geometric parameters (Å, °)

**Table:**

O1—C1	1.205 (3)	C6—H6B	0.9700
O2—C1	1.362 (3)	C7—C10	1.533 (3)
O2—C8	1.471 (3)	C7—C8	1.548 (3)
O3—C12	1.357 (3)	C8—C11	1.491 (3)
O3—C13	1.373 (4)	C8—H8	0.9800
C1—C2	1.470 (3)	C9—H9A	0.9600
C2—C3	1.337 (3)	C9—H9B	0.9600
C2—C7	1.504 (3)	C9—H9C	0.9600
C3—C4	1.506 (4)	C10—H10A	0.9600
C3—C9	1.507 (4)	C10—H10B	0.9600
C4—C5	1.518 (4)	C10—H10C	0.9600
C4—H4A	0.9700	C11—C12	1.357 (4)
C4—H4B	0.9700	C11—C14	1.415 (4)
C5—C6	1.531 (4)	C12—H12	0.9300
C5—H5A	0.9700	C13—C14	1.331 (4)
C5—H5B	0.9700	C13—H13	0.9300
C6—C7	1.526 (3)	C14—H14	0.9300
C6—H6A	0.9700		
			
C1—O2—C8	109.29 (17)	C6—C7—C8	113.2 (2)
C12—O3—C13	106.9 (3)	C10—C7—C8	111.41 (18)
O1—C1—O2	119.5 (2)	O2—C8—C11	109.30 (19)
O1—C1—C2	131.9 (2)	O2—C8—C7	103.70 (17)
O2—C1—C2	108.6 (2)	C11—C8—C7	119.00 (18)
C3—C2—C1	128.0 (2)	O2—C8—H8	108.1
C3—C2—C7	124.8 (2)	C11—C8—H8	108.1
C1—C2—C7	107.02 (18)	C7—C8—H8	108.1
C2—C3—C4	120.4 (2)	C3—C9—H9A	109.5
C2—C3—C9	124.1 (3)	C3—C9—H9B	109.5
C4—C3—C9	115.5 (2)	H9A—C9—H9B	109.5
C3—C4—C5	116.0 (2)	C3—C9—H9C	109.5
C3—C4—H4A	108.3	H9A—C9—H9C	109.5
C5—C4—H4A	108.3	H9B—C9—H9C	109.5
C3—C4—H4B	108.3	C7—C10—H10A	109.5
C5—C4—H4B	108.3	C7—C10—H10B	109.5
H4A—C4—H4B	107.4	H10A—C10—H10B	109.5
C4—C5—C6	112.6 (2)	C7—C10—H10C	109.5
C4—C5—H5A	109.1	H10A—C10—H10C	109.5
C6—C5—H5A	109.1	H10B—C10—H10C	109.5
C4—C5—H5B	109.1	C12—C11—C14	105.5 (2)
C6—C5—H5B	109.1	C12—C11—C8	123.8 (3)
H5A—C5—H5B	107.8	C14—C11—C8	130.7 (2)
C7—C6—C5	110.0 (2)	C11—C12—O3	110.2 (3)
C7—C6—H6A	109.7	C11—C12—H12	124.9
C5—C6—H6A	109.7	O3—C12—H12	124.9
C7—C6—H6B	109.7	C14—C13—O3	109.2 (2)
C5—C6—H6B	109.7	C14—C13—H13	125.4
H6A—C6—H6B	108.2	O3—C13—H13	125.4
C2—C7—C6	110.41 (19)	C13—C14—C11	108.2 (3)
C2—C7—C10	109.49 (19)	C13—C14—H14	125.9
C6—C7—C10	112.3 (2)	C11—C14—H14	125.9
C2—C7—C8	99.28 (17)		

### Hydrogen-bond geometry (Å, °)

**Table:**

*D*—H···*A*	*D*—H	H···*A*	*D*···*A*	*D*—H···*A*
C8—H8···O1^i^	0.98	2.58	3.510 (3)	158

Symmetry codes: (i) *x*−1, *y*, *z*.
